# Subtype-specific associations between breast cancer risk polymorphisms and the survival of early-stage breast cancer

**DOI:** 10.1186/s12967-018-1634-0

**Published:** 2018-10-01

**Authors:** Fangmeng Fu, Wenhui Guo, Yuxiang Lin, Bangwei Zeng, Wei Qiu, Meng Huang, Chuan Wang

**Affiliations:** 1 Breast Surgery Ward, Department of General Surgery, Fujian Medical University Union Hospital, Fuzhou, 350001 Fujian Province China; 2 Nosocomial Infection Control Branch, Fujian Medical University Union Hospital, Fuzhou, 350001 Fujian Province China; 3 Fujian Center for Disease Control and Prevention, Fuzhou, 350001 Fujian Province China

**Keywords:** Breast cancer, Single nucleotide polymorphism, Genome-wide association study, Prognosis

## Abstract

**Background:**

Limited evidence suggests that inherited predisposing risk variants might affect the disease outcome. In this study, we analyzed the effect of genome-wide association studies—identified breast cancer-risk single nucleotide polymorphisms on survival of early-stage breast cancer patients in a Chinese population.

**Methods:**

This retrospective study investigated the relationship between 21 GWAS-identified breast cancer-risk single nucleotide polymorphisms and the outcome of 1177 early stage breast cancer patients with a long median follow-up time of 174 months. Cox proportional hazards regression models were used to estimate the hazard ratios and their 95% confidence intervals. Primary endpoints were breast cancer special survival and overall survival while secondary endpoints were invasive disease free survival and distant disease free survival.

**Results:**

Multivariate survival analysis showed only the rs2046210 GA genotype significantly decreased the risk of recurrence and death for early stage breast cancer. After grouping breast cancer subtypes, significantly reduced survival was associated with the variant alleles of rs9485372 for luminal A and rs4415084 for triple negative breast cancer. Importantly, all three single-nucleotide polymorphisms, rs889312, rs4951011 and rs9485372 had remarkable effects on survival of luminal B EBC, either individually or synergistically. Furthermore, statistically significant multiplicative interactions were found between rs4415084 and age at diagnosis and between rs3803662 and tumor grade.

**Conclusions:**

Our results demonstrate that breast cancer risk susceptibility loci identified by GWAS may influence the outcome of early stage breast cancer patients’ depending on intrinsic tumor subtypes in Chinese women.

**Electronic supplementary material:**

The online version of this article (10.1186/s12967-018-1634-0) contains supplementary material, which is available to authorized users.

## Background

Breast cancer (BC) is the most common diagnosed cancer and the fifth leading cause of cancer death among women in China [1]. The 5-year survival of early stage breast cancer (EBC) patients in China is about 58–78%, which is low compared to that in American and varies in different geographic areas of China [2]. Traditionally, there are some prognostic factors for EBC survival including tumor size, lymph node involvement, tumor grade, hormone receptor (HR) status. However it has been proven that inherited host characteristics, such as single nucleotide polymorphisms (SNPs), play an important role [3].

Recently, genome-wide association studies (GWAS) have been widely applied to search genetic variations and disease association. It is worth noting that some susceptibility genes or polymorphisms identified by GWAS have been proven to not only be associated with predisposition to malignant tumors, but also influence their clinical outcome [4–6]. Only one study and one meta-analysis examined the relationship between GWAS-identified BC risk polymorphisms and the outcome for BC, both of which focused on Caucasian populations [6, 7]. However, rs6504950 and rs3803662 had different effects on the survival of BC patients in those two studies. Differences might be due to the different sample sizes and the different enrolled BC cases. Still, those studies already demonstrated the possible associations between BC risk loci and BC survival.

Similarly, there had been some BC-risk GWAS focusing on East Asian women and that found several BC risk variants, most of which were different from those identified in other ethnic populations [8, 9]. However, the relation between these polymorphisms and survival of EBC Asian patients has never been established. In the present study, we analyzed the association between 21 GWAS-identified SNPs and the survival of patients in Southeastern China with EBC.

## Methods

### Study populations

This is a hospital-based study including 1177 early breast cancer cases from Fujian Medical University Union Hospital from July 2000 and October 2014. All the participants were histopathologically confirmed with invasive breast cancer and subsequently treated with curative surgical resection and systemic therapy. Clinicopathological and demographic data were collected from the hospital records and survival data were obtained from the followed-up database which was renewed annually. The patients were staged according to the 7th version of American Joint Commission on Cancer (AJCC) tumor-node-metastasis (TNM) staging system [10]. Estrogen receptor (ER)/progesterone receptor (PR) positivity was determined by IHC analysis of the number of positively stained nuclei (≥ 10%) and hormone receptor (HR) positivity was defined as being either ER+ and/or PR+. Tumors were considered human epidermal growth factor-2 (HER2) positive when cells exhibited strong membrane staining (3+). Expressions of 2+ would require further in situ hybridization testing for HER2 gene amplification while expressions of 0 or 1+ were regarded as negative. The subtypes were categorized as follows [11]: luminal A (ER+, PR+ > 20%, HER2−, Ki67 < 14% or grade I when Ki67 was unavailable), luminal B (HR+, HER2−, Ki67 > 14% or grade II/III when Ki67 was unavailable or HR+, HER2+); HER2 enriched (HR−, HER2+) and triple negative (HR− and HER2−). The study was approved by the Institutional Ethics Committee and all participants consented to genetic testing at the time of their participation and contributed data.

### SNPs selection

We selected the polymorphisms associated with breast cancer susceptibility from the US National Human Genome Research Institute (NHGRI) Catalog of Published Genome-Wide Association Studies. We used the following inclusion criteria: (i) the significance level for genome-wide association was considered to be *P *≤ 1 × 10^−9^; (ii) the minor allele frequency (MAF) was at least 10% in the HapMap CHB data of the public SNP database (http://www.ncbi.nlm.nih.gov/SNP); (iii) pair wise linkage disequilibrium (LD) between the eligible SNPs calculated by Haploview 4.1 software must be less than 0.8 (*r*^2^ < 0.8). At last, 21 polymorphisms were applied in this study which can be found in Additional file 1: Table S1.

### DNA extraction and SNPs genotyping

Blood samples were collected in EDTA anticoagulant tubes and stored at − 80 °C until DNA extraction. Genomic DNA was extracted using the Whole-Blood DNA Extraction Kit (Bioteke, Beijing, China), according to the manufacturer’s protocol. The genotype analysis was performed by SNPscan, which is a high-throughput SNPs genotyping technology (Genesky Biotechnologies Inc., Shanghai, China). Finally, the raw data were analyzed by the GeneMapper 4.0 Software (Applied Biosystems, Foster City, CA). 5% of samples were randomly selected as blinded duplicates for quality assessment purposes and 100% concordance was obtained.

### Statistical analyses

Overall survival (OS) and breast cancer specific survival (BCSS) were our primary endpoints and defined as the time from the date of cancer diagnosis to the date of mortality for all cause and breast cancer, respectively. Disease free survival (DFS) and distant disease free survival (DDFS) were our secondary endpoints and calculated separately as the time from the date of diagnosis to the date of any recurrence and distant recurrence to the last patient contact [12]. Survival data were analyzed using the Kaplan–Meier method with the log-rank test and multivariate Cox stepwise regression analysis to the end of follow-up (2016.12.31). Adjustment for age at diagnosis, tumor size, lymph node involvement, histological grade, ER status, and HER-2/neu expression were applied. The hazard ratios (HRs) and 95% confidence interval (CI) for each factor in multivariate analyses were calculated from the Cox-regression model. The Chi square-based Q test was used to examine the heterogeneity between subgroups. The possible gene-environment interactions were also evaluated by the Cox proportional hazard regression models. All tests were 2-sided, and P values of < 0.05 were considered statistically significant. SAS 9.4 (SAS Institute Inc., Cary, NC) was used for all statistical analyses.

## Results

### Patient characteristics and clinical features

Patients’ clinical characteristics and survival are summarized in Table 1. All the 1177 early breast cancer cohort, were female and their mean age was 47.0 ± 10.3 years old at breast cancer diagnosis. During a median follow-up time of 174 months, 446 cases experienced recurrence (142 locoregional and 410 distant) and 343 died (333 died of BC and 10 died of other disease).

**Table 1:** Patients’ clinicopathological characteristics and clinical outcome

Variables	Patients N = 1177	iDFS		DDFS		BCSS		OS	
		Events	LogRank *P*	Events	LogRank *P*	Events	LogRank *P*	Events	LogRank *P*
Age at diagnosis			0.021		0.087		0.420		0.402
≤ 35	184	85		76		59		61	
> 35	993	361		334		274		282	
Tumor size (cm)			< 0.001		< 0.001		< 0.001		< 0.001
≤ 2	403	88		80		67		70	
> 2	774	358		330		266		273	
Nodal status			< 0.001		< 0.001		< 0.001		< 0.001
Negative	510	116		101		69		75	
Positive	667	330		309		264		268	
Clinical stage			< 0.001		< 0.001		< 0.001		< 0.001
I	257	40		35		29		31	
II + III	920	406		375		304		312	
Grade^a^			< 0.001		< 0.001		< 0.001		< 0.001
I + II	904	310		286		228		236	
III	271	134		122		103		105	
ER			< 0.001		< 0.001		< 0.001		< 0.001
Negative	378	177		165		149		150	
Positive	799	269		245		184		193	

Variables	Patients N = 1177	iDFS	DDFS	BCSS	OS	iDFS	DDFS	BCSS	OS
		Events	LogRank *P*	Events	LogRank *P*	Events	LogRank *P*	Events	LogRank *P*
PR			< 0.001		< 0.001		< 0.001		< 0.001
Negative	367	171		159		144		145	
Positive	810	275		251		189		198	
HER2			< 0.001		< 0.001		< 0.001		< 0.001
Negative	860	292		268		214		222	
Positive	317	154		142		119		121	
Subtype			< 0.001		< 0.001		< 0.001		< 0.001
Luminal A	236	35		33		26		26	
Luminal B	574	240		218		163		172	
HER2+	160	80		76		67		67	
Triple negative	207	91		83		77		78	

^a^Variable including missing data

No significant difference in BC-DDFS, BCSS, and OS was shown in the subgroup of age at diagnosis (*P* = 0.087, 0.420, and 0.402). But patients with a tumor size > 2 cm, lymph node positive, grade III, clinical stage II + III, or HER2 positive had significantly shorter survival times, whereas being ER or HR positivity remarkably improved the survival of EBC patients (log-rank P < 0.05, Table 1). Furthermore, our intrinsic molecular subtypes (luminal A, luminal B, HER2-enriched, and triple negative) were also associated with significantly different survival (log-rank P < 0.05, Table 1).

### Effects of each polymorphism on survival of EBC

Among the 21 SNPs, 6 SNPs (rs13281615, rs4415084, rs4784227, rs889312, rs10474352 and rs10816625) had a log-rank P under 0.05 in some genetic models and in some outcome indicators (log-rank P < 0.05, Table 2). But after adjusting for age at breast cancer diagnosis, tumor size, lymph node involvement, grade, hormone receptor status, and HER2 status, only rs889312 and rs2046210 had significant effect on improving survival of EBC patients. In a recessive model, rs889312 was significantly associated with better iDFS and DDFS (iDFS: adjusted HR (aHR): 0.761, 95% CI 0.583–0.994, and DDFS: aHR: 0.631, 95% CI 0.470–0.848; Table 3). Similarly, in contrast to the GG + AA genotypes, the GA genotype of rs2046210 also improve the survival of EBC patients (iDFS: aHR: 0.812, 95% CI 0.673–0.980; DDFS: aHR: 0.771, 95% CI 0.635–0.938; BCSS: aHR: 0.790, 95% CI 0.636–0.981 and OS aHR: 0.786, 95% CI 0.635–0.934, Table 3).

**Table 2:** Genotyping results with EBC’s survival

SNPs	Cases WH/H/VH	iDFS (LogRank *P*)				DDFS (LogRank *P*)				BCSS (logRank *P*)				OS (Log Rank *P*)			
		Events WH/H/VH	DOM	REC	COD	Events WH/H/VH	DOM	REC	COD	Events WH/H/VH	DOM	REC	COD	Events WH/H/VH	DOM	REC	COD
rs10069690	789/353/34	298/139/8	0.938	0.065	0.152	273/129/8	0.689	0.128	0.221	218/107/7	0.510	0.230	0.291	225/110/7	0.533	0.191	0.257
rs13281615	293/575/308	126/196/124	0.043	0.397	0.035	112/186/112	0.178	0.619	0.241	86/154/93	0.592	0.402	0.482	89/157/97	0.531	0.320	0.362
rs13387042	932/234/11	351/91/4	0.803	1.000	0.968	322/84/4	0.767	0.830	0.944	264/66/3	0.891	0.891	0.977	274/66/3	0.664	0.934	0.898
rs1562430	801/344/32	297/136/13	0.419	0.787	0.720	272/125/13	0.363	0.516	0.600	228/97/8	0.840	0.738	0.938	234/100/9	0.940	0.955	0.996
rs2046210	361/602/214	142/220/84	0.327	0.873	0.611	134/198/78	0.180	0.964	0.361	107/162/64	0.359	0.970	0.633	112/166/65	0.231	0.829	0.481
rs2180341	715/394/68	270/147/29	0.858	0.381	0.679	245/136/29	0.556	0.136	0.326	198/115/20	0.554	0.783	0.836	204/118/21	0.547	0.676	0.809
rs2981582	493/545/139	187/204/55	0.891	0.459	0.708	173/189/48	0.843	0.843	0.945	143/149/41	0.491	0.554	0.556	146/154/43	0.581	0.459	0.547
rs3112612	776/354/46	290/140/15	0.541	0.563	0.610	263/132/14	0.263	0.660	0.391	210/110/12	0.213	0.818	0.393	218/111/13	0.290	1.000	0.545
rs3803662	532/512/133	214/185/47	0.102	0.472	0.258	193/172/45	0.309	0.795	0.594	157/138/38	0.284	0.957	0.537	165/139/39	0.141	0.946	0.309
rs4415084	392/558/226	144/204/98	0.332	0.043	0.124	130/189/91	0.256	0.038	0.106	105/152/76	0.245	0.039	0.107	110/156/77	0.345	0.059	0.160
rs4784227	550/513/113	191/211/44	0.035	0.714	0.104	177/195/38	0.077	0.905	0.164	146/155/32	0.173	0.793	0.389	148/162/33	0.091	0.773	0.235
rs889312	346/631/200	130/252/64	0.770	0.059	0.111	124/235/51	0.823	0.003	0.010	98/189/46	0.840	0.070	0.142	101/196/46	0.841	0.038	0.080
rs9485372	388/588/200	136/227/82	0.122	0.177	0.200	127/208/74	0.230	0.360	0.415	104/169/59	0.334	0.529	0.592	107/173/62	0.320	0.382	0.513
rs10474352	374/572/230	158/214/74	0.052	0.029	0.041	143/199/68	0.142	0.049	0.101	115/161/57	0.285	0.156	0.301	119/165/59	0.241	0.160	0.284
rs10816625	350/595/231	145/213/88	0.047	0.825	0.127	136/196/78	0.022	0.468	0.073	114/156/63	0.017	0.559	0.056	118/160/65	0.012	0.567	0.041
rs12922061	539/529/108	199/206/41	0.590	0.905	0.865	185/188/37	0.799	0.926	0.953	156/147/30	0.613	0.943	0.877	158/154/31	0.847	0.970	0.981
rs2290203	270/587/319	96/229/121	0.464	0.891	0.760	89/211/110	0.519	0.962	0.800	67/174/92	0.218	0.689	0.468	69/179/95	0.206	0.647	0.449
rs2296067	418/567/191	160/215/71	0.869	0.814	0.968	144/200/66	0.774	0.923	0.940	116/166/51	0.649	0.751	0.798	119/172/52	0.590	0.668	0.704
rs2981578	416/548/212	150/219/77	0.465	0.619	0.556	132/208/70	0.148	0.512	0.650	105/172/56	0.158	0.488	0.166	110/176/57	0.232	0.421	0.210
rs4951011	522/528/126	204/191/51	0.350	0.421	0.340	186/178/46	0.516	0.475	0.516	150/142/41	0.597	0.163	0.233	157/145/41	0.388	0.246	0.230
rs9693444	572/486/118	215/179/52	0.762	0.154	0.357	196/164/50	0.619	0.068	0.188	156/141/36	0.379	0.483	0.616	160/144/39	0.329	0.259	0.428

*WH/H/VH* wide homozygous type/heterozygote/variant homozygous type, *DOM* dominant model, *REC* recessive model, *COD* codominant model

**Table 3:** Association between the SNPs’ genotype with EBC’ survival (multivariate cox proportional hazard model)

SNPs	Cases	iDFS			DDFS			BCSS			OS		
		Events	Adjusted HR (95% CI)^a^	*P* value	Events	Adjusted HR (95% CI)^a^	*P* value	Events	Adjusted HR (95% CI)^a^	*P* value	Events	Adjusted HR (95% CI)^a^	*P* value
All cases													
rs889312													
CC	346	130	1 (reference)		124	1 (reference)		98	1 (reference)		101	1 (reference)	
CA	631	252	1.089 (0.880–1.347)	0.433	235	1.065 (0.856–1.326)	0.569	189	1.087 (0.850–1.389)	0.507	196	1.094 (0.859–1.393)	0.465
AA	200	64	0.804 (0.595–1.087)	0.157	51	0.658 (0.474–0.913)	0.012	46	0.814 (0.573–1.158)	0.253	46	0.782 (0.510–1.111)	0.170
DOM			1.017 (0.828–1.248)	0.876		0.960 (0.777–1.187)	0.706		1.020 (0.804–1.293)	0.872		1.017 (0.805–1.285)	0.887
REC			0.761 (0.583–0.994)	0.045		0.631 (0.470–0.848)	0.002		0.772 (0.564–1.055)	0.105		0.738 (0.540–1.009)	0.057
rs2046210													
GG	361	142	1 (reference)		134	1 (reference)		107	1 (reference)		112	1 (reference)	
GA	602	220	0.796 (0.644–0.985)	0.035	198	0.761 (0.610–0.949)	0.015	162	0.775 (0.606–0.991)	0.042	166	0.762 (0.598–0.970)	0.027
AA	214	84	0.948 (0.722–1.244)	0.700	78	0.963 (0.727–1.275)	0.792	64	0.951 (0.696–1.299)	0.752	65	0.919 (0.675–1.250)	0.589
DOM			0.833 (0.682–1.018)	0.074		0.809 (0.658–0.996)	0.045		0.818 (0.649–1.031)	0.090		0.800 (0.638–1.005)	0.055
REC			1.094 (0.861–1.391)	0.462		1.142 (0.890–1.464)	0.296		1.116 (0.847–1.469)	0.436		1.089 (0.829–1.430)	0.541
OVE			0.812 (0.673–0.980)	0.030		0.771 (0.635–0.938)	0.009		0.790 (0.636–0.981)	0.033		0.786 (0.635–0.934)	0.028
Luminal A													
rs9485372													
GG	72	10	1 (reference)		10	1 (reference)		7	1 (reference)		7	1 (reference)	
GA	124	16	0.833 (0.372–1.863)	0.656	14	0.717 (0.313–1.644)	0.432	11	0.890 (0.332–2.385)	0.817	11	0.890 (0.332–2.385)	0.817
AA	40	9	2.201 (0.883–5.486)	0.090	9	2.192 (0.880–5.459)	0.092	8	3.280 (1.152–9.378)	0.026	8	3.280 (1.152–9.378)	0.026
DOM			1.087 (0.518–2.283)	0.825		0.995 (0.469–2.109)	0.989		1.328 (0.546–3.229)	0.532		1.328 (0.546–3.229)	0.532
REC			2.465 (1.133–5.360)	0.023		2.671 (1.214–5.875)	0.015		3.522 (1.464–8.473)	0.005		3.522 (1.464–8.473)	0.005
Triple negative													
rs4415084													
TT	59	24	1 (reference)		20	1 (reference)		20	1 (reference)		20	1 (reference)	
CT	83	44	1.622 (0.979–2.688)	0.061	42	1.799 (1.048–3.087)	0.033	39	1.686 (0.975–2.917)	0.062	40	1.736 (1.006–2.996)	0.047
CC	65	23	1.785 (0.996–3.201)	0.052	21	1.813 (0.971–3.385)	0.062	18	1.549 (0.809–2.969)	0.187	18	1.551 (0.810–2.972)	0.186
DOM			1.674 (1.043–2.687)	0.033		1.804 (1.084–3.002)	0.023		1.640 (0.979–2.750)	0.060		1.674 (1.000–2.803)	0.049
REC			1.345 (0.827–2.187)	0.232		1.274 (0.765–2.120)	0.352		1.139 (0.661–1.962)	0.639		1.119 (0.650–1.926)	0.685
Luminal B													
rs4951011													
AA	265	120	1 (reference)		109	1 (reference)		82	1 (reference)		88	1 (reference)	
GA	253	92	0.682 (0.526–0.896)	0.006	84	0.698 (0.524–0.929)	0.014	59	0.652 (0.466–0.914)	0.013	62	0.630 (0.454–0.874)	0.006
GG	55	28	0.883 (0.579–1.346)	0.562	25	0.888 (0.568–1.386)	0.645	22	1.025 (0.631–1.664)	0.921	22	0.965 (0.597–1.560)	0.885
DOM			0.719 (0.557–0.928)	0.011		0.734 (0.561–0.960)	0.024		0.721 (0.528–0.984)	0.039		0.690 (0.510–0.934)	0.016
REC			1.068 (0.714–1.598)	0.749		1.075 (0.703–1.645)	0.738		1.259 (0.794–1.998)	0.328		1.205 (0.762–1.908)	0.425
rs889312													
CC	162	74	1 (reference)		70	1 (reference)		51	1 (reference)		54	1 (reference)	
CA	308	135	1.304 (0.778–1.374)	0.819	126	1.048 (0.782–1.406)	0.753	94	1.113 (0.790–1.568)	0.542	100	1.108 (0.794–1.546)	0.545
AA	104	31	0.570 (0.373–0.870)	0.009	22	0.432 (0.266–0.701)	0.001	18	0.534 (0.310–0.918)	0.023	18	0.498 (0.290–0.853)	0.011
DOM			0.901 (0.684–1.187)	0.459		0.871 (0.654–1.160)	0.344		0.954 (0.682–1.333)	0.781		0.940 (0.679–1.301)	0.708
REC			0.558 (0.381–0.817)	0.003		0.419 (0.269–0.653)	< 0.000		0.498 (0.304–0.815)	0.006		0.465 (0.285–0.761)	0.002
Luminal B													
rs9485372													
GG	204	72	1 (reference)		63	1 (reference)		47	1 (reference)		49	1 (reference)	
GA	275	125	1.439 (1.076–1.924)	0.014	115	1.524 (1.121–2.073)	0.007	89	1.517 (1.065–2.162)	0.021	93	1.520 (1.075–2.149)	0.018
AA	95	43	1.622 (1.111–2.370)	0.122	38	1.665 (1.116–2.485)	0.013	27	1.463 (0.910–2.350)	0.116	30	1.596 (1.012–2.516)	0.044
DOM			1.482 (1.124–1.954)	0.005		1.557 (1.161–2.088)	0.003		1.504 (1.071–2.112)	0.018		1.538 (1.104–2.142)	0.011
REC			1.307 (0.939–1.820)	0.112		1.294 (0.914–1.831)	0.146		1.137 (0.752–1.720)	0.544		1.239 (0.835–1.839)	0.288

*DOM* dominant model, *REC* recessive model, *OVE* overdominant model

^a^*HR* hazard risk, *CI* confidence interval; For all patients: Adjusted for age at diagnosis, tumor size, lymph node involvement, grade, hormone receptor status and Her2 status; For subtypes: Adjusted for age at diagnosis, tumor size, lymph node involvement, grade

### Prognostic implication of risk variants in molecular subtypes

For a large number of patients enrolled in this study, we analyzed the association between enrolled SNPs and survival associated with different molecular subtypes of EBC. As showed in Table 3, rs9485372 and rs4415084 were still associated with a worse outcome in luminal A and triple negative EBC patients, respectively, after adjustment (for rs9485372 under the recessive model: iDFS: aHR: 2.465, 95% CI 1.133–5.360; DDFS: aHR: 2.671, 95% CI 1.214–5.875; BCSS and OS: aHR: 3.522, 95% CI 1.464–8.473; for rs4415084 under the dominant model: iDFS: aHR: 1.674, 95% CI 1.043–2.687; DDFS: aHR: 1.804, 95% CI 1.084–3.002 and OS: aHR: 1.674, 95% CI 1.000–2.803). Furthermore, in the luminal B subtype we found that rs4951011 (under the dominant model) and rs889312 (under the recessive model) could significantly improve the iDFS, DDFS, BCSS and OS of the breast cancer, while rs9485372 (under dominant model) worsens outcome (iDFS: aHR = 0.719, 95% CI 0.557–0.928, DDFS: aHR = 0.734, 95% CI 0.561–0.960, BCSS: aHR = 0.721, 95% CI 0.528–0.984 and OS: aHR = 0.690, 95% CI 0.510–0.934 for rs4951011; iDFS: aHR = 0.558, 95% CI 0.381–0.817, DDFS: aHR = 0.419, 95% CI 0.269–0.653, BCSS: aHR = 0.498, 95% CI 0.304–0.815 and OS: aHR = 0.465, 95% CI 0.285–0.761 for rs889312 and iDFS: aHR = 1.482, 95% CI 0.124–1.954, DDFS: aHR = 1.557, 95% CI 0.161–2.088, BCSS: aHR = 1.504, 95% CI 1.071–2.112 and OS: aHR = 1.538, 95% CI 1.104–2.142 for 9485872, Table 3). However, no significant effect was observed in the HER2-enriched subtype in any model of the 21 polymorphisms.

### Combined analysis of three risk SNPs on survival of luminal B EBC

**Fig. 1:**
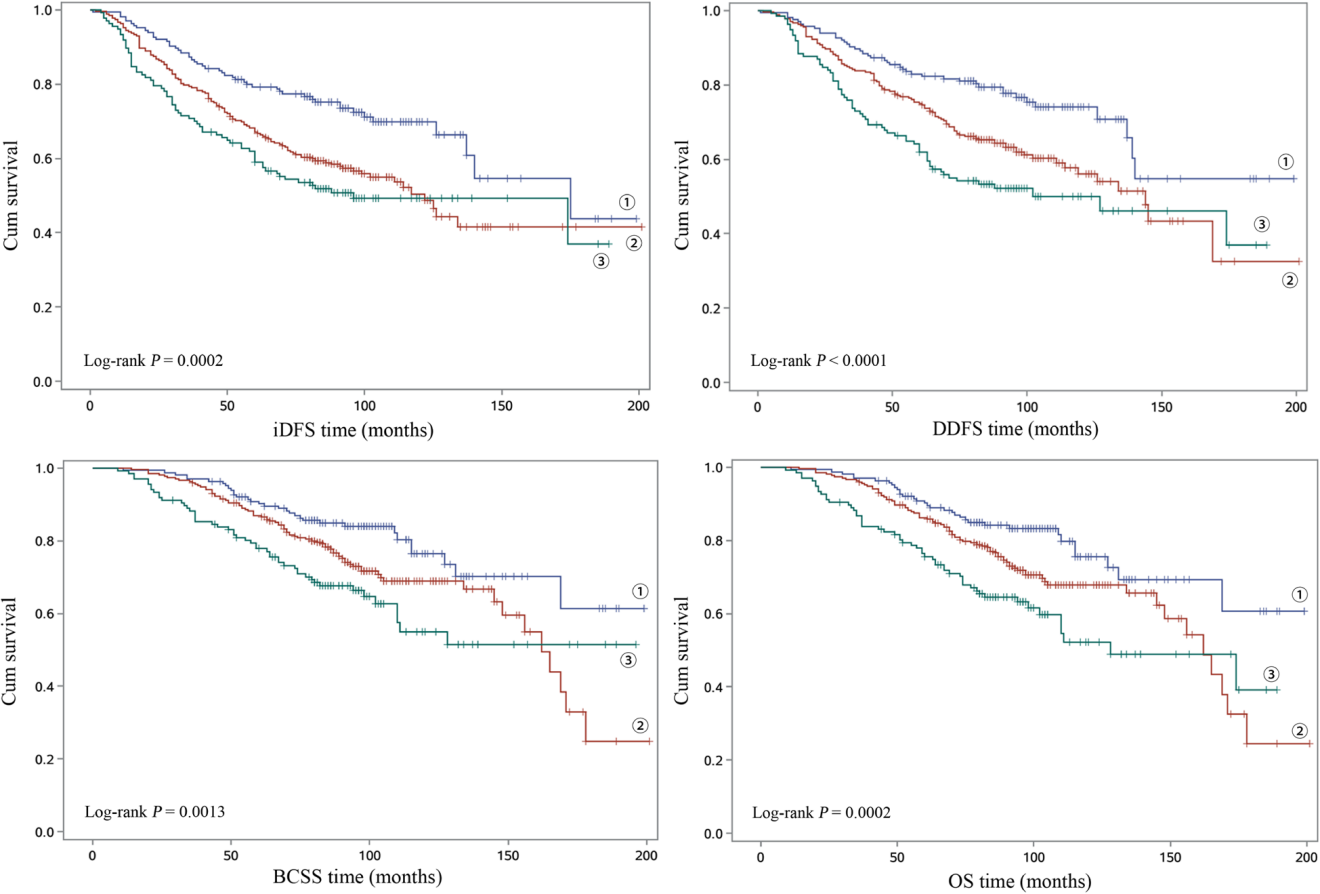
Kaplan–Meier plots of survival for combined effect of the three SNPs on luminal B EBC survival

To assess the combined effects on risk of recurrence and death from luminal B EBC, we combined the risk genotypes of rs4951011, rs889312 and 9485372. According to the number of combined risk genotypes, the univariate survival analysis show that all of iDFS, DDFS, BCSS and OS were significantly different among different groups with different combined risk genotypes (*P* Log-rank < 0.01) (Fig. 1). As shown in Table 4, compared to subjects with one or no unfavorable genotype, subjects carrying more unfavorable loci had shorter survival time and had a 1.534–1.645 fold increased risk of recurrence and/of death even after adjustment (iDFS: aHR = 1.534, 95% CI 1.288–1.827, DDFS: aHR = 1.632, 95% CI 1.356–1.964, BCSS: aHR = 1.570, 95% CI 1.267–1.944 and OS: aHR = 1.645, 95% CI 1.334–2.029, respectively for trend).

**Table 4:** Cumulative effect of unfavorable genotypes in luminal B subtype breast cancer

Number of risk genotypes^a^	Cases	iDFS			DDFS			BCSS			OS		
		Events	Adjusted HR (95% CI)^b^	*P* value	Events	Adjusted HR (95% CI)^b^	*P* value	Events	Adjusted HR (95% CI)^b^	*P* value	Events	Adjusted HR (95% CI)^b^	*P* value
0–1	165	49	1 (reference)		42	1 (reference)		32	1 (reference)		33	1 (reference)	
2	272	123	1.912 (1.369–2.670)	1.44 × E−4	109	1.894 (1.324–2.711)	4.74 × E−4	81	1.787 (1.184–2.697)	5.70 × E−3	84	1.786 (1.192–6.678)	4.97 × E−3
3	137	68	2.431 (1.679–3.519)	2.52 × E−6	67	2.744 (1.862–4.043)	3.53 × E−7	50	2.525 (1.617–3.943)	4.61 × E−5	55	2.755 (1.786–4.251)	4.59 × E−6
Trend *P*			1.534 (1.288–1.827)	1.63 × E−6		1.632 (1.356–1.964)	2.18 × E−7		1.570 (1.267–1.944)	3.66 × E−5		1.645 (1.334–2.029)	3.25 × E−6

^a^rs4951011 AA, rs889312 CC + CA and rs9485372 GA + AA were presumed as unfavorable genotypes

^b^*HR* hazard risk, *CI* confidence interval; Adjusted for age at diagnosis, tumor size, lymph node involvement, grade

### Stratification and interaction analysis

The associations between breast cancer risk loci genotypes and EBC survival were then evaluated by stratified analysis of age at diagnosis, tumor size, lymph node involvement, grade, hormone-receptor status and HER2 status. As shown in Table 5, we found that rs4415084 and rs2981582 were associated with shorter survival of the patients who were younger (rs4415084 for age at diagnosis ≤ 35 years: iDFS: aHR = 1.792, 95% CI 1.161–2.915, DDFS: aHR = 2.172, 95% CI 1.310–3.602, BCSS: aHR = 2.250, 95% CI 1.278–3.959 and OS: aHR = 1.871, 95% CI 0.988–3.544) and with higher grade tumors (rs2981582 for grade III: iDFS: aHR = 1.666, 95% CI 1.051–2.639, DDFS: aHR = 1.682, 95% CI 1.049–2.698, BCSS: aHR = 1.783, 95% CI 1.080–2.944 and OS: aHR = 1.732, 95% CI 1.050–2.855). But rs2046210 and rs3803662 had beneficial effects on survival of the patients with larger tumor (rs2046210 for tumor size > 2 cm: iDFS: aHR = 0.757, 95% CI 0.606–0.944, DDFS: aHR = 0.732, 95% CI 0.582–0.919, BCSS: aHR = 0.713, 95% CI 0.533–0.920 and OS: aHR = 0.694, 95% CI 0.540–0.992) and with higher grade tumors (rs3803662 for grade III: iDFS: aHR = 0.588, 95% CI 0.414–0.834, DDFS: aHR = 0.586, 95% CI 0.407–0.845, BCSS: aHR = 0.479, 95% CI 0.319–0.717 and OS: aHR = 0.484, 95% CI 0.324–0.722) respectively. However, we did not find that the other SNPs affected survival in the subgroups of patients with different tumor characteristics.

**Table 5:** Stratification analysis of polymorphism genotypes associated with EBC survival

SNPs	Variables	iDFS		DDFS		BCSS		OS	
		Adjusted HR (95% CI)	*P* value^a^	Adjusted HR (95% CI)	*P* value^a^	Adjusted HR (95% CI)	*P* value^a^	Adjusted HR (95% CI)	*P* value^a^
rs4415084	Age at diagnosis								
	≤ 35	1.792 (1.161–2.915)	0.068	2.172 (1.310–3.602)	0.014	2.250 (1.278–3.959)	0.018	1.871 (0.988–3.544)	0.009
	> 35	1.073 (0.830–1.386)		1.056 (0.809–1.379)		1.067 (0.796–1.431)		0.743 (0.584–0.946)	
rs2046210	Tumor size (cm)								
	≤ 2	1.277 (0.791–2.061)	0.052	1.277 (0.773–2.109)	0.048	1.558 (0.874–2.780)	0.015	1.522 (0.867–2.670)	0.012
	> 2	0.757 (0.606–0.944)		0.732 (0.582–0.919)		0.713 (0.553–0.920)		0.694 (0.540–0.992)	
rs2981582	Grade								
	I + II	0.922 (0.642–1.323)	0.048	0.791 (0.532–1.177)	0.017	0.822 (0.529–1.278)	0.023	0.872 (0.571–1.331)	0.040
	III	1.666 (1.051–2.639)		1.682 (1.049–2.698)		1.783 (1.080–2.944)		1.732 (1.050–2.855)	
rs3803662	Grade								
	I + II	1.017 (0.812–1.273)	0.010	1.096 (0.866–1.387)	0.005	1.151 (0.884–1.500)	0.000	1.075 (0.830–1.392)	0.001
	III	0.588 (0.414–0.834)		0.586 (0.407–0.845)		0.479 (0.319–0.717)		0.484 (0.324–0.722)	

Adjusted for age at diagnosis, tumor size, lymph node involvement, grade, hormone receptor, HER2 status, exception for stratification factor

*HR* hazard risk, *CI* confidence interval

^a^Heterogeneity test for differences between groups

An interaction analysis was performed (Table 6) and statistically significant multiplicative interactions on EBC survival were found both between rs4415084 genotypes and age at diagnosis (adjusted *P*int: iDFS 0.045, DDFS 0.013, BCSS 0.025 and OS 0.018) and between rs3803662 genotypes and tumor grade (adjusted *P*int: iDFS 0.011, DDFS 0.001, BCSS 4.7 × 10^−4^ and OS 9.9 × 10^−4^).

**Table 6:** The interaction analysis between risk variants and clinicopathological parameters

SNPs	Variable	iDFS		DDFS		BCSS		OS	
		Adjusted HR^a^	*P* value	Adjusted HR^a^	*P* value	Adjusted HR^a^	*P* value	Adjusted HR^a^	*P* value
rs4415084	Age at diagnosis								
CC	≤ 35	1 (reference)		1 (reference)		1 (reference)		1 (reference)	
CC	> 35	1.113 (0.739–1.676)	0.609	1.270 (0.814–1.983)	0.292	1.366 (0.829–2.249)	0.221	1.346 (0.827–2.189)	0.232
CT	≤ 35	1.317 (0.797–2.176)	0.282	1.421 (0.829–2.438)	0.202	1.358 (0.733–2.516)	0.331	1.271 (0.692–2.336)	0.440
CT	> 35	1.090 (0.734–1.619)	0.669	1.246 (0.810–1.917)	0.316	1.373 (0.847–2.229)	0.198	1.340 (0.835–2.148)	0.225
TT	≤ 35	2.013 (1.161–3.488)	0.013	2.427 (1.357–4.339)	0.003	2.505 (1.310–4.788)	0.005	2.497 (1.328–4.693)	0.004
TT	> 35	1.180 (0.767–1.815)	0.452	1.332 (0.836–2.124)	0.228	1.461 (0.868–2.460)	0.153	1.378 (0.826–2.298)	0.219
*P* for multiplicative interaction			0.045		0.013		0.025		0.018
rs3803662	Grade								
GG	I + II	1 (reference)		1 (reference)		1 (reference)		1 (reference)	
GG	III	1.858 (1.400–2.466)	1.8E−5	1.877 (1.394–2.527)	3.3E−5	2.134 (1.543–2.952)	4.6E−6	2.018 (1.469–2.773)	1.5E−5
GA	I + II	1.031 (0.814–1.306)	0.801	1.106 (0.864–1.416)	0.425	1.139 (0.862–1.505)	0.361	1.054 (0.801–1.385)	0.709
GA	III	1.043 (0.746–1.459)	0.804	1.014 (0.711–1.446)	0.939	0.979 (0.655–1.462)	0.917	0.946 (0.639–1.403)	0.784
AA	I + II	0.994 (0.684–1.443)	0.973	1.081 (0.735–1.592)	0.691	1.246 (0.820–1.893)	0.303	1.195 (0.793–1.800)	0.394
AA	III	1.085 (0.582–2.023)	0.798	1.245 (0.665–2.331)	0.493	1.043 (0.501–2.169)	0.911	0.983 (0.474–2.041)	0.964
*P* for multiplicative interaction			0.011		0.001		4.7E−4		9.9E−4

^a^*HR* hazard risk, *CI* confidence interval; adjusted for age at diagnosis, tumor size, Lymph node involvement, grade, hormone receptor status and HER2 status, except for the interaction factor

## Discussion

In this study, we evaluated the possible relation between 21 GWAS-identified BC susceptibility germline variations and EBC clinical outcome in a large Chinese cohort of 1177 EBC cases. To the best of our knowledge, this is the first study that reports the association between GWAS-identified BC susceptibility loci and clinical outcomes in a Chinese population and it produced different results from two other American studies findings [6, 7]. The most significant and novel result of this study is that the influence of BC risk polymorphisms on the outcome of EBC depends on different intrinsic molecular subtypes, especially for luminal B breast cancer.

More recently, Zhang and his colleagues demonstrated some GWAS-identified SNPs are associated with molecular subtypes of EBC in Chinese women [13]. It has been accepted worldwide that breast cancer is a complex disease and consists of several intrinsic subtypes, which have different etiologies and prognosis [14]. By altering the related genes’ expression and/or function in key signaling pathways, we gradually realize putative SNPs may take effect on the basis of molecular subtypes, whether in risk or in clinical outcome of EBC [15–17].

Loci rs889312, rs4951011, and rs9485372 play significant and independent roles in survival of luminal B breast cancer patients both individually or jointly by all of the four outcome indicators (iDFS, DDFS, BCSS and OS). Recently, MAP3K1 rs889312 has been identified as a low-penetrant risk factor for breast cancer, both for ER+ or ER− breast cancer [18]. It was also demonstrated to be an independent risk factor for poor survival in diffuse-type gastric cancer in an overdominant model [19]. However, two similar investigations failed to prove this variant was associated with BC clinical outcome [6, 7], although neither of them carried out survival analysis on the basis of BC intrinsic subtypes. From most recent available data, rs889312 (C/C) was found to be significantly associated with poor DFS, DDFS and OS among HR positive breast cancer patients [20], which was similar to our results. The MAP3K1 gene is the most important member in the MAPK signal pathway which activates the transcription of essential cancer genes [21]. But the exact mechanism as to how rs889312 can change MAP3K1 protein structure and/or function is still beyond our knowledge.

The rs4951011 located in intron 2 of the zinc finger CCCH domain-containing protein 11A (ZC3H11A) and 5′-UTR of ZBED6 gene, has been first identified as a BC susceptibility loci in East Asian [8]. In another study, it was only associated with triple negative breast cancer but not other BC subtypes [22]. For rs4951011 in the dominant model, we found that the GA + GG genotype was significantly associated with a better DFS, DDFS, BCSS and OS (aHR = 0.690–0.734). However, there was no evidence indicating a relation between this variant and clinical outcome of other malignant tumors. The data of ENCODE from human mammary epithelial cells (HMEC) suggests that rs4951011 may be located in a strong enhancer region marked by peaks of several active histone acetylation modifications (H3K4me1, H3K4me3, H3K9ac, and H3K27ac) [23]. Furthermore, it was found in colorectal cancer cell lines that repressing transcription of ZBED6 modulates expression of 10 genes, including PTBN1, WWC1, WWTR1, etc., linked to important signal pathway and tumor development depended on the genetic background of tumor cells and the transcription state of its target genes [24]. So rs4951011 may regulate expression of some important metastasis-related genes and then influence the course of breast cancer.

The SNP rs9485372 was also found to play a significant role in the clinical outcome of luminal A and luminal B breast cancer patients. For luminal A BC, rs9485372 in the recessive model had a worse iDFS, DDFS, BCSS, and OS (aHR 2.465–3.522). For luminal B BC, the GA + AA genotypes had a worse iDFS, DDFS, BCSS and OS (aHR = 1.482–1.557), compared to the GG genotype. This variant is located in Table 2  (TGF-β activated kinase 1/MAP3K7 binding protein 2) which plays a pivotal role in the TGF-β pathway and contributes to development of cancer [25]. Table 2 is near the ESR1 gene and it was found to be co-expressed with ESR1 in hepatocellular carcinoma [26]. Table 2 was found to be a mediator of resistance to endocrine therapy which is a poor prognostic indicator for HR+ breast cancer patients and is a potential new target to reverse pharmacological resistance and potentiate anti-estrogen action [27]. Therefore it is possible that the association both rs9485372 and survival of luminal A and B BC patients may be mediated by regulating estrogen signaling and the TGF-β pathway.

Two GWAS-identified BC risk loci, rs1219648 and rs13387042, were found to take effect on overall survival of EBC in Tunisians [28]. On the contrary, we failed to confirm this result in our Chinese population. We attribute this difference to the following reasons. Firstly, these two studies focused on different ethnic groups with different genetics background. Secondly, we used a much bigger sample size and longer follow-up than the other study which made our result more reliable. Finally, both of these two studies are retrospective. We used the multivariate Cox proportional hazard model to evaluate the independent effect of every SNP on survival of EBC patients while the other study just used Kaplan–Meier Curve and Log-Rank Test.

Some potential limitations of our study should be taken into consideration. First, as all patients were of Chinese origin, it is unclear whether our findings are Chinese Han population—specific or common in other populations. Second, the biological mechanism of the significant SNPs in breast cancer is still unclear. Therefore, more studies with diverse ethnic backgrounds and determination of the functional characterizations of the SNPs are warranted. Nevertheless, this is the first study with integrated clinicopathological data and long enough follow-up data to investigate the association between genetic breast cancer risk polymorphisms and survival of Asian breast cancer patients depended on intrinsic molecular subtypes.

## Conclusions

Our findings indicated that breast cancer risk variants are not in general strongly associated with clinical outcome. However, we illustrated that, on the basis of molecular subtypes, there are some potential BC risk polymorphisms, which are probably novel predictors for EBC outcome in Chinese patients. Large better-designed investigations with a variety of populations, as well as functional assessments are needed to verify and extend our findings.

## Additional file

**Additional file 1: Table S1.** Information about of the breast cancer risk SNPs identified by GWAS applied in our study

## Abbreviations

GWAS: genome-wide association study
SNPs: single nucleotide polymorphisms
BC: breast cancer
EBC: early-stage breast cancer
HRs: hazard ratios
CIs: confidence intervals
BCSS: breast cancer special survival
OS: overall survival
iDFS: invasive disease free survival
DDFS: distant disease free survival
HR: hormone receptor
AJCC: American Joint Commission on Cancer
TNM: tumor-node-metastasis
ER: estrogen receptor
PR: progesterone receptor
HER2: human epidermal growth factor-2
MAF: minor allele frequency
NHGRI: National Human Genome Research Institute
ZC3H11A: zinc finger CCCH domain-containing protein 11A
HMEC: human mammary epithelial cells
