# Supplementary material for: Subtype-specific associations between breast cancer risk polymorphisms and the survival of early-stage breast cancer
Source: J Transl Med. 2018 Oct 1;16:270. doi: 10.1186/s12967-018-1634-0 (PMC6167771; doi:10.1186/s12967-018-1634-0)
Supplement: Supplementary file 1 — Additional file 1: Table S1. Information about of the breast cancer risk SNPs identified by GWAS applied in our study [file 12967_2018_1634_MOESM1_ESM.docx]

Table S1: Information about of the breast cancer risk SNPs identified by GWAS applied in our study

| SNPs | Genes | Base change  Major/Minor allel | Location ^a^ | Coding annotation ^b^ | Genotyping rate | MAF in  cases | Reference |
| --- | --- | --- | --- | --- | --- | --- | --- |
| rs4951011^c^ | ZC3H11A/ZBED6 | A/G | Chr1:203766331 | Intronic variant | 99.90% | 0.334 | 1 |
| rs13387042 | LOC101928278 | A/G | Chr2:217905832 | Intronic variant | 100% | 0.109 | 2 |
| rs10069690 | TERT | C/T | Chr5:1279790 | Intronic variant | 99.90% | 0.179 | 3 |
| rs4415084 | MRPS30 | C/T | Chr5:44662515 | intergenic | 99.90% | 0.429 | 2 |
| rs889312 | MAP3K1 | A/C | Chr5:56031884 | Intronic variant | 100% | 0.438 | 4 |
| rs10474352^c^ | ARRDC3 | C/T | Chr5:90732225 | intergenic | 99.90% | 0.439 | 1 |
| rs2180341 | RNF146 | A/G | Chr6:127600630 | Intronic variant | 100% | 0.225 | 5 |
| rs9485 372^c^ | TAB2 | G/A | Chr6:149608874 | Intronic variant | 99.90% | 0.424 | 6 |
| rs2046210^c^ | ESR1 | A/G | Chr6:151948366 | intergenic | 100% | 0.438 | 7 |
| rs13281615^c^ | CASC21, CASC8 | A/G | Chr8:128355618 | intergenic | 99.90% | 0.494 | 1 |
| rs1562430 | FAM84B | T/C | Chr8:128387852 | intergenic | 100% | 0.173 | 8 |
| rs9693444 | C8orf75 | C/A | Chr8:29509616 | intergenic | 99.90% | 0.307 | 9 |
| rs10816625 | LOC105376214 | A/G | Chr9:110837073 | intergenic | 99.90% | 0.45 | 10 |
| rs2296067 | KDM4C | G/A | Chr9:6984236 | Missense variant | 99.90% | 0.403 | 11 |
| rs2981578^c^ | FGFR2 | C/T | Chr10:123340311 | Intronic variant | 99.90% | 0.413 | 12 |
| rs2981582 | FGFR2 | G/A | Chr10:493545139 | Intronic variant | 100% | 0.35 | 4 |
| rs2290203^c^ | PRC1 | C/T | Chr15:91512067 | Intronic variant | 99.90% | 0.479 | 1 |
| rs4784227^c^ | TNRC9/TOX3 | C/T | Chr16:52599188 | Intronic variant | 99.90% | 0.331 | 6 |
| rs12922061 | 16q12/TOX3 | C/T | Chr16:52635000 | Intronic variant | 99.90% | 0.317 | 13 |
| rs3112612 | TNRC9/TOX3 | A/G | Chr16:52635164 | Intronic variant | 99.90% | 0.19 | 13 |
| rs3803662 | TNRC9/TOX3 | G/A | Chr16:52586341 | Intronic variant | 100% | 0.331 | 8 |

^a^  Genome Reference Consortium Human, build 37 (http://genome.ucsc.edu/cgi-bin/hgGateway)

^b^ Coding annotations for these SNPs were retrieved from the 1000 genomes (http://browser.1000genomes.org/index.html) and dbSNP (http://www.ncbi.nlm.nih.gov/SNP/)

^C^ These SNPs are proved to be associated with breast cancer risk in Asian populations.

**References**

1. Cai Q, Zhang B, Sung H,et al. Genome-wide association analysis in East Asians identifies breast cancer susceptibility loci at 1q32.1, 5q14.3 and 15q26.1. Nat Genet. 2014; 46: 886-90. <http://www.ncbi.nlm.nih.gov/pubmed/25038754.>
2. Stacey SN, Manolescu A, Sulem P, et al. Common variants on chromosomes 2q35 and 16q12 confer susceptibility to estrogen receptor-positive breast cancer. Nat Genet. 2007; 39:865-9. http://www.ncbi.nlm.nih.gov/pubmed/17529974.
3. Haiman CA, Chen GK, Vachon CM,et al. A common variant at the TERT-CLPTM1L locus is associated with estrogen receptor-negative breast cancer. Nat Genet. 2011; 43: 1210-4. http://www.ncbi.nlm.nih.gov/pubmed/22037553.
4. Easton DF, Pooley KA, Dunning AM, et al. Genome-wide association study identifies novel breast cancer susceptibility loci. Nature. 2007; 447:1087-93. http://www.ncbi.nlm.nih.gov/pubmed/17529967.
5. Gold B, Kirchhoff T, Stefanov S, Lautenberger J, et al. Genome-wide association study provides evidence for a breast cancer risk locus at 6q22.33. Proc Natl Acad Sci U S A. 2008; 105: 4340-4345.http://www.ncbi.nlm.nih.gov/pubmed/18326623.
6. Long J, Cai Q, Shu XO, et al. Identification of a functional genetic variant at 16q12.1 for breast cancer risk: results from the Asia Breast Cancer Consortium. PLoS Genet. 2010, 6: e1001002.http://www.ncbi.nlm.nih.gov/pubmed/20585626.
7. Zheng W, Long J, Gao YT, Li C, Zheng Y, Xiang YB, Wen W, Levy S, Deming SL, Haines JL, Gu K, Fair AM, Cai Q, Lu W, Shu XO. Genome-wide association study identifies a new breast cancer susceptibility locus at 6q25.1. Nat Genet. 2009; 41: 324-28. [http://www.ncbi.nlm.nih.gov/ pubmed/](http://www.ncbi.nlm.nih.gov/pubmed/)19219042.
8. Turnbull C, Ahmed S, Morrison J, et al. Genome-wide association study identifies five new breast cancer susceptibility loci. Nat Genet. 2010; 42: 504-7.http://www.ncbi.nlm.nih.gov/pubmed/20453838.
9. Michailidou K, Hall P, Gonzalez-Neira A, et al. Large-scale genotyping identifies 41 new loci associated with breast cancer risk. Nat Genet. 2013; 45: 353-61. http://www.ncbi.nlm.nih.gov/pubmed/23535729.
10. Orr N, Dudbridge F, Dryden N, et al. Fine-mapping identifies two additional breast cancer susceptibility loci at 9q31.2. Hum Mol Genet. 2015; 24: 2966-84. http://www.ncbi.nlm.nih.gov/pubmed/25652398.
11. Hong Q, Yu S, Yang Y, Liu G, Shao Z. A polymorphism in JMJD2C alters the cleavage by caspase-3 and the prognosis of human breast cancer.

Oncotarget. 2014; 5: 4779-87. http://www.ncbi.nlm.nih.gov/pubmed/24952432.

Hunter DJ, Kraft P, Jacobs KB, et al. A genome-wide association study identifies alleles in FGFR2 associated with risk of sporadic postmenopausal breast cancer. Nat Genet. 2007; 39: 870-4.http://www.ncbi.nlm.nih.gov/pubmed/17529973.

1. Low SK, Takahashi A, Ashikawa K, Inazawa J, Miki Y, Kubo M, Nakamura Y, Katagiri T. Genome-wide association study of breast cancer in the Japanese population. PLoS One. 2013; 8: e76463.http://www.ncbi.nlm.nih.gov/pubmed/24143190.
2. Rinella ES, Shao Y, Yackowski L, et al. Genetic variants associated with breast cancer risk for Ashkenazi Jewish women with strong family histories but no identifiable BRCA1/2 mutation. Hum Genet. 2013; 132: 523-36. http://www.ncbi.nlm.nih.gov/pubmed/23354978.
